# Lateralised Behavioural Responses in Livestock to Environmental Stressors: Implications for Using Infrared Thermography to Assess Welfare Conditions

**DOI:** 10.3390/ani13233663

**Published:** 2023-11-27

**Authors:** Amira A. Goma, Jashim Uddin, Emily Kieson

**Affiliations:** 1 Department of Animal Husbandry and Animal Wealth Development, Faculty of Veterinary Medicine, Alexandria University, Alexandria 21944, Egypt; 2 Department of Veterinary and Animal Sciences, University of Raishahi, Rajshahi 6205, Bangladesh; jashimvet2008@ru.ac.bd; 3 Department of Research, Equine International, Boston, MA 02120, USA; ekieson@equineintl.org

**Keywords:** lateralisation, infrared thermography, behavioural responses, stressors, livestock, welfare conditions

## Abstract

**Simple Summary:**

As concerns over animal welfare grow, individuals working in animal husbandry need easier ways to assess the psychological wellbeing of livestock. Recent research in lateralisation and stress has helped scientists find links between stress and the sides of the body animals use to inspect new objects and people or engage in new experiences. Research has shown that the left side of the brain processes familiar conditions, while the right side of the brain is more often used for new situations. These sides of the brain correspond to behaviours expressed with the opposite side of the body. Animals often use a specific eye or leg when initiating behavioural responses or interactions which can be assumed to link to the opposite side of the brain. This article summarised the existing literature for lateralisation in common livestock species in addition to advocating for increased use of thermography when assessing welfare in livestock.

**Abstract:**

Lateralised behavioural responses to environmental stressors have become more frequently used as indicators of social welfare in animals. These lateralised behavioural responses are under the control of asymmetrical brain functions as part of the primary functions of most vertebrates and assist in primary social and survival functions. Lateralised behavioural responses originating from the left hemisphere are responsible for processing familiar conditions, while the right hemisphere is responsible for responding to novel stimuli in the environment. The forced lateralisation and side preference tests have been used to determine the visual lateralised behavioural responses in livestock to environmental stressors. Limb preference during movement has also been used to determine motor lateralisation. Although behavioural investigations in livestock have recorded lateralised behavioural responses to environmental stressors, there are still limitations in the implication of lateralisation to other conditions, such as restraint and invasive procedures. Thus, it is important to have a non-invasive measure for these lateralised behavioural responses. Recently, lateralised behavioural responses have been correlated with the use of infrared temperature of external body surfaces, such as the eyes and coronary bands of limbs. This review summarised the different forms of the lateralised behavioural responses in livestock, especially cattle and horses, to environmental stressors, and the association between these responses and the relevant external body surfaces’ infrared temperature, with the purpose of improving the use of non-invasive measures in assessing welfare conditions in animals. The combination of the lateralised behavioural responses and infrared temperature of external body surfaces to environmental stressors could improve the assessment strategies of welfare conditions and the related additional husbandry interventions that could be applied to improve the welfare of farm animals.

## 1. Introduction

The assessment of the cognitive, mental, and emotional welfare of animals is increasing in importance [1,2,3,4,5], as formal welfare assessments are mainly based on animals’ health [6,7]. In addition to traditional assessment strategies, several studies have given particular attention to the validation of lateralised behaviours as being welfare indicators of the animals’ cognitive and emotional welfare. For example, Knierim et al. [8] stated that behavioural responses that are related to high proportions of fluctuating cerebral asymmetry could indicate poor welfare. Therefore, understanding the process of the lateralised behavioural visuospatial responses to a challenging environment could help to avoid the suffering and distress of animals [9,10,11]. 

Lateralisation means asymmetry of body functions [12], which enhances neural processing through the reduction of conflict and interference that improves task performance [13]. Examples of these are the left- and right-handedness in humans and limb preferences in animals [14,15,16]. The fundamental and complementary patterns of brain hemispheric lateralisation in vertebrates include the involvement of the right hemisphere in rapid responses and the left hemisphere in the control of motor responses that require some degree of initial inhibition before a final decision is made. For example, the preference of using the right forelimb (i.e., left hemisphere) by vertebrates when fine motor manipulation is required to be performed [17].

Environmental stimulation has an important role in the development of brain lateralisation [18]. The exposure of animals to environmental stimulation in their early life enhances brain lateralisation that could prepare them to live in a world that requires good cognitive ability. For example, Lyons et al. [19] found that intermittent separations of Saimiri monkeys from their mothers led to more stimulation, which, in turn, leads to the development of a lateralised brain. Conversely, lower environmental stimulation links to less variable brain function and redundancy (symmetrical/non lateralised) and thus fewer demands on the animal’s cognitive ability. For example, light deprivation during early stages of chick development has shown to impede the development of brain lateralisation. Hens normally block light from reaching the eggs during incubation but occasionally leave their nest, exposing eggs to light for short durations, which then leads to brain lateralisation in the developing chick. Light deprivation in chicks at this early developmental stage could potentially indicate a poor living environment, which would then lead to brain redundancy [18].

Lateralisation of behavioural responses and the connection with brain laterality has been studied in a variety of species, specifically where the lateralisation is associated with stress [20,21]. Stress comes in two forms: acute and chronic. Acute stress stimulates the hypothalamus to secrete more vasopressin than corticotropin-releasing hormone, whereas chronic stress has the reverse effect and is often associated with prolonged high levels of cortisol. Animals’ ability to run or fight is elevated when growth hormones and blood sugar levels are affected, which enables them to react to acute stimuli quickly. Chronic stress, on the other hand, leads to long-term consequences, like stunted growth and heightened illness vulnerability [22]. This chronic stress (and corresponding prolonged high levels of cortisol) influences the lateralisation of behaviours and can therefore become an indicator of welfare for animals. For example, intensively housed cattle, who are reared almost entirely indoors (with limited pasture access), showed more lateralised responses than those with continuous access to pasture (with indoor housing at night) [23]. Stub et al. [24] reported increased evidence of lateralised leg use in rats housed individually on a grid floor when compared to those housed socially on bedding. Moreover, Tuyttens et al. [25] found that rabbits housed in enriched and low-density cages exhibited fewer lateralised responses than those housed in barren and high-density cages. 

Furthermore, studies have shown that lateralised behavioural responses reflect how animals overcome specific emotional conditions, facilitating the classification of emotions via valence and arousal [26,27]. These can be studied by blocking the sensory organs in the left side and subsequently the right side and comparing the differences in lateralised behavioural responses [28]. These have also been studied using specialised tests like the detour test for visual responses [29], the head-turn test for auditory responses [30], and the orienting asymmetry paradigm [31]. 

The role of lateralisation has also been studied with regards to stress and social interactions within the domestic environment. The evolution of social behaviours is related to the development of higher cognitive abilities [32] and the ability of individuals to distinguish between conspecifics. For example, researchers have found that *Bos taurus* cattle could distinguish between conspecifics in addition to specific visual discrimination [33]. In the same context, Vallortigara and Rogers [13] found that social interactions were also lateralised within species. This specialised lateralisation helps an individual to avoid being attacked by another through approaching the latter on its right side, thus avoiding its antagonistic left side (i.e., right hemisphere). Therefore, social recognition was found to be processed in a consistent pattern in vertebrates, with the right hemisphere responsible for individual recognition and the left hemisphere responsible for distinctions between conspecifics and heterospecifics [34].

Most research on lateralisation has focused on behavioural approaches, but other studies have demonstrated that the lateralisation of other actions can be used to determine preferences and lateralised stress responses. For example, researchers found that hyperthermia could be induced when the animal was under acute stress conditions [35] due to the activation of the sympathetic nervous system, causing blood to be moved from the periphery to the vital organs [36,37]. The change in peripheral blood provides an opportunity to measure temperature variations and physiological changes that correlate with stress. However, common methods of measuring this temperature change required handlers to restrain animals to apply the temperature detection sensors, potentially causing irritation and distress in the animals [38,39,40,41,42]. 

More recently, additional technology has been developed to determine these temperature changes without restraint or distress to animals. These new approaches could then be used to indicate physiological and psychological changes in animals. Infrared thermography is one of the most common forms of non-invasive approaches in measuring peripheral blood flow and temperature as a matter of detecting stress in animals. The process of infrared thermography depends on the amount of infrared energy an animal emits from its exterior. This technique uses a thermal camera to measure peripheral energy, translates that energy into temperature, and then displays the temperature distribution as an image of the thermogram (a picture of pixels with different colours or shades representing different infrared temperatures) [43,44]. This technique has been used to measure the peripheral temperature changes in cattle [45,46,47] and in horses [48,49,50]. For example, in cattle, eye infrared temperature was found to decline under aversive handling conditions [51], surgical castration [52], and infusion of epinephrine [53]. Furthermore, the nasal temperature also decreased in response to negative emotional states in dairy cows [37,54]. In horses, the infrared temperature of the eye has been successfully used to determine stress levels in response to different sporting performances [55], human handling [50,56], and interaction with riding equipment [57]. Contrary to the cattle and horses, the infrared temperature of eyes in rabbits and dogs has been found to elevate due to stress. This discrepancy may be due to the differences in the time of measurements between infrared thermography and the beginning of the exposure to the stressor [58,59]. 

More recently, a new dimension of assessing animal welfare has been developed which focuses on the relationship between the lateralised behavioural responses and the infrared temperature of the animal’s external body surfaces. The infrared temperatures of eyes and limbs were of particular interest in the identification of anxiety (right side/right hemisphere preference) in dairy cows [60,61]. This increasing knowledge regarding lateralisation in farm animals via its various forms [62], in addition to its association with infrared thermography, can help in better understanding the cognitive functions of these animals and allows for the development of new approaches for welfare improvement [63,64,65]. 

## 2. Relationships between Sensory Lateralisation and Behavioural Responses

Animals’ behavioural responses are dependent upon how they perceive and understand their environment [66]. As part of their learning, sensory cues are processed as either positive or negative stimuli, which assist animals in seeking or avoiding pleasant or unpleasant environmental consequences [67,68]. For example, dairy cows learn to enter a milking parlour by associating auditory cues with food rewards [69]. Also, domestic ungulates use their sensory cues to discriminate between ‘friends and foes’ and, accordingly, decrease or increase their level of vigilance [70]. For example, cows decreased their vigilance and increased foraging rates for the high-quality forages when they detected olfactory or visual cues from a heterospecific species placed near the high-quality food. The opposite responses occurred when a predator species was placed near the high-quality food [70].

Sensory lateralisation is a subfield of lateralisation in which the expression of lateralisation in the visual, olfactory, and auditory senses changes in mammals and humans during particular situations. These are controlled via hemispheric specialisation, as mentioned above. Rogers [64] proposed that the preferences of the use of a specific eye, ear, and nostril can help in understanding cognitive processes. Also, identification of the particular eye, ear, and nostril involved in the response can represent how the animal is processing its environmental stimuli. This lateralised preference also indicates an effect on emotional responses [71]. 

### 2.1. Visual Lateralisation

Domestic livestock ungulates have specific visual fields related to the placement of their eyes (on the sides of their heads) that affect their behaviours with regards to how they orientate themselves to stimuli. Knowledge regarding these animals’ visual fields of view can therefore provide additional information to the importance of visual lateralisation and behavioural responses to environmental changes. For example, cows depend on vision for about 50% of their information. Their eyes are located on the sides of their head, so they have a wide field of view (about 330 degrees). However, they have a limited binocular field of vision about 30–50 degrees, with a blind spot directly behind them [72]. Like cattle, horses have eyes on the sides of their heads and are able to detect and discriminate between stimuli in an almost full 360-degree circle around them, including a panoramic field of view [73].

Vertebrates and non-vertebrate mammals have little or no overlapping of the visual fields of view (sided eyes). This means that these types of mammals process their visual information in a lateralised way [74,75]. These mammals employ lateralised behavioural patterns to assess their surroundings by adjusting their head position and using eye movements to indicate lateralisation [29]. The fundamental process of the lateralised vision therefore depends on the connection of the left field of vision to the right brain hemisphere and vice versa. 

These perceptual asymmetries have been observed and measured in daily management routines. In their daily lives, animals must make choices regarding their daily routines, providing researchers some insight into their stress and emotional responses based on the lateralisation of behaviours during these decision-making processes. Fraser and Matthews [76] reported that such preferences aid in assessing what could be important for animals, thus improving their welfare. For example, horses displayed right side visual preference for novel objects (neutral emotional valence), left eye preference for objects with negative valence, and no side preference for objects with positive valence (suggesting binocular vision was used) [71]. Furthermore, under new environments, horses often use the left eye to assess conditions, especially when a human is present, and also showed left eye preference when assessing or scanning a novel human [77]. Since horses are traditionally trained to accept humans on the left side, horses in this particular study were trained to accept a person on both eyes to alleviate side bias [77].

### 2.2. Auditory Lateralisation

Much like visual lateralisation, auditory lateralisation is also important in assessing welfare and analysing behavioural responses in animals. Auditory senses are much more sensitive in cattle in comparison to humans. Cattle perceive a wider range of frequencies (from 23 to 37,000 Hz), with a maximum sensitivity of 8000 Hz [78]. These hearing frequencies enable them to detect predators at greater distances and to locate the source of noises [79]. This trait also enables them to hear and identify their own kind. For example, a calf can recognise its mother’s calls and differentiate them from others [78]. Furthermore, cattle move their ears upwards to listen carefully and constantly while remaining vigilant, then localise the noise source via the auricle (external ear). This ability is effective when the noise comes from the front of the animal, but it is reduced to 25° if the noise is at the sides of the animals’ heads [80]. Therefore, cattle must turn their heads to identify the noise source. 

Andrew [81] reported that the head and ear orientations in mammals, called the “orientation reflex”, are considered an external clue for the arousal state of the animal. In this sense, the arousal state can be determined by observing changes in head orientation of the passive unrestrained individuals in response to the playback of vocalisation of familiar conspecifics from behind. These changes in orientation have also been used for examining lateralised responses to acoustic stimuli in mammals tested under unrestrained contexts [82]. The direction of the head turning indicates the role of the contralateral hemisphere in the acoustic stimulus processing. For example, an individual turns its head while the right ear is directed towards the speaker to process inputs from the right ear via the left hemisphere [82,83]. Although the turning of the head in many species does not allow the observer to distinguish between visual and auditory lateralisation, this can still be determined by retraining the head and only allowing the ears to move in response to sound [84]. Under these circumstances, individual animals can direct their ears and/or head either independently or simultaneously towards the sound source and demonstrate laterality and neurological processing. 

Auditory lateralisation has been studied in a variety of species in which the hemispheric specialisation is affected by various factors, such as sound structure, species, and type of stimulus [85]. However, researchers have also found a large degree of duplication in hemispheric function in mammals during experiments with auditory stimuli, where each ear connects to both brain sides due to varies decussations and commissures beginning in the medulla [86]. In this case, each hemisphere receives signals from both ears, and then processes them and makes comparisons between the right and left ear inputs without the intervention of the other hemisphere. During this processing, one hemisphere could localise sound at any point in the horizontal plane or decode speech/sound entering either ear, even in the absence of cerebral commissures [87]. Therefore, the auditory signal can be processed predominantly in the contralateral hemisphere, leading to asymmetrical processing even if one ear is connected to both hemispheres [88]. Thus, if researchers observe an animal turn its head to the left or move the left ear backwards towards the sound, this indicates control via the right hemisphere and vice versa [83]. This is consistent with findings that the vocalisations of conspecifics were reported to be processed within the left hemisphere [89]. 

In other studies, cattle’s sense of hearing was reported to determine its behaviour on the farm as they adapted to familiar noises. This is true even for novel, intense, or high-pitched sounds that may lead to fearful reactions [90]. However, this is not the same for low-pitched sounds, which tend to soothe the animal [90]. In addition, voice inflections in people and the degree of familiarity of the voice can lead to behavioural changes [91]. For example, the sound of a human cry (scream) can lead to more expressed agitation in cattle than a metallic sound. Moreover, Waiblinger et al. [92] stated that cattle respond to variations in human vocalisation ranging from soothing sounds to signals indicating danger that resulted in behaviours indicating fear, aggression, or uneasiness. Furthermore, cattle seem to dislike shouting more than aversive physical contact [93]. A similar phenomenon has also been observed in horses. Auditory responses in horses have shown that they use the left hemisphere (right ear) to process vocalisation from horses that they recognise, but are outside of their social group, and the right hemisphere (left ear) to process vocalisation from unknown horses [85]. 

### 2.3. Olfactory Lateralisation

Most mammalian species can detect and discriminate different odours and pheromones through olfaction. There are differences not only between species but also individuals in their olfactory sensitivity. Most researchers studying the olfactory sensitivity in mammals depend on behavioural testing [94]. For example, stressed cattle, or its urine odour, can modify the behavioural reactions of its conspecifics [95], like slowing the learning ability in heifers [96]. Moreover, a longer latency to start feeding in addition to a slower feeding rate were observed in cattle approaching an object contaminated with the urine of stressed conspecifics. This indicates increased fearfulness, which is mediated through olfactory signals from the pheromones in the urine of distressed animals [95]. Therefore, pheromones can be considered as an alarm signal to herd members. 

In addition to non-human animals, olfactory responses were also reported to be lateralised in humans [97], in which the right nostril (i.e., right hemisphere) was found to respond to unfamiliar odours [98]. However, in another study by Broman et al. [99], it was reported that the right nostril also responded more strongly than the left nostril to familiar odours. However, in mammals, an ipsilateral ascent to the olfactory system due to the information’s transmit from each nostril to the olfactory cortex via the olfactory bulb of the corresponding hemisphere was reported [100]. Previous studies have deducted the lateralised processing of odour analysis in both vertebrate and invertebrate species [71,100,101,102,103], and it was reported that the response to novel information was under the right hemispheric control, while the left hemisphere controlled familiar information [74,101,104,105,106]. Therefore, the response of the right nostril (i.e., right hemisphere) to adrenaline was in line with the idea that the right hemisphere controls the hypothalamic–pituitary–adrenal axis, which is associated with the expression of arousal as well as negative emotions, such as aggression, escape behaviours, and fear [101]. Therefore, sympathetic activation was under the control of the right hemisphere, while the left hemisphere controlled the parasympathetic activity that is associated with calm responses [107]. However, olfactory lateralisation in horses has shown that there was a slight, but not significant, tendency to use the right nostril to investigate novel objects [71]. 

## 3. Relationship between Sensory Behavioural Lateralisation and Emotions

The link between emotions and behavioural lateralisation resulted in increased awareness of livestock stakeholders regarding enhanced adaptive fitness and welfare during regular handling of their animals. For example, during emotional conditions, visual lateralisation in cattle was observed by Robins and Phillips [9], in which the left/right eye was turned towards the emotional stimulus to process it in the contralateral brain hemisphere, a response that was considered a lateralisation measure. They recorded that 97 cattle used the left eye and 53 used the right eye to see the given negative emotional stimulus at the time of crossing the transect path. Therefore, the right hemisphere is related to the left eye that allows an animal to view the potential threat more easily [9]. Further, Siniscalchi et al. [108] revealed that visual stimuli with higher emotional valence (negative) also resulted in animals turning to the left side due to the contralateral brain structure’s activation.

Additionally, in another study, Robins and Phillips [9] investigated the eye preferences of a cohort of cattle who were familiar with the stimuli. This group were then exposed to the same negative emotional stimuli as the ones previously mentioned, which resulted in a demonstration of a reversal of viewing preferences. Out of 72 cattle who crossed the transect path, 29 cattle used the left eye, but 43 cattle used the right eye to observe the stimuli. This directional shift in viewing preferences indicated experience-dependent learning or habitual effects on emotional and behavioural lateralisation, which has also been observed in other mammalian and non-mammalian animal species. 

Phillips et al. [10] observed that the cows that preferred to use their left eye to view dangerous situations were lower in the dominance order and had increased crush scores. In this study, they had demonstrated behaviours such as moving down the left-hand side when they became familiar with the “danger”, so they were assumed to be more anxious cows. Furthermore, Kappel et al. [109] investigated the relationship between preferential uses of the left or right eye and other behaviours while passing unfamiliar bilaterally placed objects while exiting the milking parlour. They reported that a higher number of cows approached the right object than the left. Cows that approached the left object (i.e., left eye/right hemisphere) exhibited hesitant behaviours, such as stopping at a distance and sniffing. Goma et al. [110] also reported that cows passing a person in the lane on their right side (i.e., left eye/right hemisphere) showed more anxious behaviours, such as sniffing to the ground, raised/tucked tail, higher crush score, and increased flight speed, than that of the cows passing on the left side. This is consistent with the study published by the authors of [64], who reported that the fight or flight response of an animal is mainly controlled via the right hemisphere of the brain. Therefore, the positive relationship between negative emotional stimulus and left eye view (i.e., right hemisphere) confirmed that the emotional stimuli processing was lateralised. 

Lateralisation has been tied to behavioural responses in horses as well. In a study of feral-living horses, researchers observed that horses had a left-side bias for agonistic and aggressive behaviours within their normal social groups as well as between stallions of different herds [111]. Horses in the same study were also observed to display left-side bias when displaying behaviours consistent with vigilance (assessing potential threat), as well as side movements of reactivity with relation to the potential threat, suggesting a preference to use the right brain hemisphere when assessing or reacting to aggression or novelty. However, these side biases did not carry over to movement [111]. 

Further studies have linked emotional valence with lateralisation in horses. In a study of young (one-year-old) domestic horses, individual horses who were approached from the left side by a novel human exhibited higher negative responses than those approached from the right. Slightly older (two-year-old) trained horses displayed no asymmetry. Since horses are often trained on their left side, and are therefore desensitised to stimuli on that side, research has suggested that the left side is more associated with negative or novel stimuli [112]. Furthermore, horses with low impulse control, indicating high emotional responses (high baseline faecal cortisol level), with innovative problem-solving capabilities who were considered tenacious also showed left-side laterality preference [113]. In another study, horses that were exposed to novel stimuli and wearing heartrate monitors displayed a greater use of their left side (right hemisphere), suggesting that the left side is important when making emotional decisions [114]. In yet another study, horses showed preference for using the left lateralisation for visual and olfactory information input when assessing stimuli associated with a negative emotional valence and no visible asymmetry when assessing stimuli with a positive valence [71]. 

Lateralisation also plays a role in social behaviours with favourite partners. Under normal conditions, horses demonstrate a left-side preference for affiliative behaviours and social interactions with favoured conspecifics [115]. During allogrooming, a social tactile interaction between two horses in which each horse simultaneously scratches the other with their teeth, horses stand next to one another facing opposite directions so that they may simultaneously favour the right or left side closest to their bonded conspecific. Recent findings have suggested that when expressing allogrooming behaviours under stressful conditions, horses do not demonstrate side preferences for this kind of social interaction [116], suggesting that while lateralisation plays a role in processing information, it may not play as strong a role when individuals are seeking affiliative interactions. Although horses and cattle have been shown to have a right hemisphere dominance pattern for processing novel or fear-inducing stimuli on a population level, there is no significant difference at the individual level, which may be a result of the tradition of training and handling domestic horses from the left side [117].

The response to vocalisations can also reflect the emotional states and serve a crucial role in the emotional contagion, which could be measured through behavioural observation of the lateralised motor expressions such as ear postures [110,118,119]. 

Nickel et al. [120] reported an asymmetrical movement of the ears in cattle, while Schmied et al. [121] observed pendulous ears of cattle during grooming, indicating that they are emotionally aroused and/or positively valenced. Additionally, De Oliveira and Keeling [122] predicted that observing the right ear positioned backwards (i.e., asymmetrical right/right ear lateralisation) indicates a positive valence. This prediction is accepting the lateralisation pattern for emotions, as the right side of the body is under the control of the left hemisphere, where the left hemisphere processes positive emotions, such as those of food rewards [117]. In addition, the left ear backwards position (i.e., asymmetrical left/left ear lateralisation) indicates negative emotions in cows which has also been studied in different species, such as sheep, horses, cats, and rats, showing the activation of the right hemisphere under stressful situations [123]. Also, Siniscalchi et al. [30] reported the involvement of the right ear in the processing of conspecific vocalisations and the left ear in the processing of threatening stimuli. Additional studies have also reported the essential role of the amygdala in the encoding of the olfactory stimuli with affective value [124,125,126]. The lateralisation of the response to stimuli of different valences in the amygdala also indicated that the left amygdala is responsible for positive stimuli and the right for the negative stimuli [127,128].

## 4. Relationship between Lateralisation and Behavioural Responses

Limb preference by livestock in locomotion has received increased attention. This limb movement preference was found to be under the control of the motor cortex in the contralateral hemisphere [13], and the lateralised limb preference has been demonstrated in the T-maze and detour test for cattle [129,130]. The detour task (or directional bias) refers to the direction in which an animal turns around a barrier. This directionality has been used to assess lateralisation preferences in various species [131]. The consistency in the direction of movement around the obstacle can be a result of the success of the first choice [132]. 

Lateralisation of movement in horses is continuing to grow as a means of assessing the cognitive and emotional responses. Horses often naturally express a tendency to favour one side or the other when grazing or moving [133], indicating individual variations in movement preference. The preferences of horses to choose a side from which they can explore the stimuli has also been studied with regard to potential “optimism”. Individual horses who preferred using the right lateral movement to initiate exploration were more likely to approach new objects in a shorter period of time, suggesting that right-dominant horses are more likely to be more positive (optimistic) [134].

Lateralisation assessments have also been employed in cognitive tasks. For example, horses expressed left-side preferences when assessed in cognitive tasks [135], and horses with predominantly left-side preference in both sensory and motor behaviours exhibited better problem-solving behaviours and cognitive innovation than those with right-sided preference [113]. Motor lateralisation has often been tested through the involvement of obstacles. When horses choose to navigate around an obstacle, they displayed a slight preference for the left-sided navigation, but these lateralisation preferences disappeared as the complexity of the task increased, suggesting that non-lateralised horses may also have better problem-solving capabilities [132].

Determining natural motor lateralisation in horses can be complicated due to their history with people. Traditionally, horses are often trained to be led and mounted from the left side, which can impact how they respond to humans and human’s interactions, including husbandry, handling, and riding. There is evidence that the tendency to favour the left lateralisation in movement increases with age [136], which could be a function of increased human interaction from the left side. The same is true for how different breeds and ages of horses laterally respond to objects given their history of training [137]. Horses naturally have a lateralisation preference which has been shown to be more left-sided preference in domestic horses, more so than in feral horses, which, in addition to being a function of handling, could also indicate increased emotional distress under these conditions [138]. Furthermore, even while carrying a passive rider (no active riding aids by the rider), horses showed that the act of carrying a person influences their motor laterality choice, but not the sensory laterality when responding to a novel stimulus [139]. Thus, the possibility that some lateralisation could be due to physical growth and genetics, as well as the evidence of biomechanical manifestations of laterality, is limited [140].

Therefore, it is important to understand animals’ directional biases which can help in understanding their preferences and their way of interaction with their environment that subsequently highlights their abilities and limitations in the environment [141]. 

## 5. Relationship between Posture Lateralisation and Behavioural Responses

Lateralisation in the posture of animals has shown to be contradictory in studies. For example, although the lying time in lactating dairy cattle was reported by the authors of [142] to be equally divided between the left and right sides, Tucker et al. [62] reported that pregnant non-lactating cows showed a preference for the left side, but there was no difference in the lying side preference in cattle in mid and late lactation. Despite these contradictory findings, a shift in the patterns of lying can occur. The preference for lying on the left side had been documented in cattle, in which the authors of [143] showed that the percentage of lying time on the left side was higher than the right side in cows and heifers (cows: 64.7 ± 1.1%, and heifers: 61.8 ± 2.7%). However, Bao and Giller [144] stated that cows change their sides of lying by about 50–60%. They change their side of lying when the previous lying bout duration increased. Therefore, when there was discomfort from the prolonged lying in one position, they change their side of lying, suggesting that changing sides may alleviate the discomfort. The probability of changing sides in the lying cattle was 50–55% when the previous lying bouts lasted less than 60 min, but this was raised to over 70% when the bouts lasted 80 min or longer. The interval between two successive lying bouts extends to 3 h until the effect of the previous lying bout diminished the ability to determine which side the cow would lie on next. These results indicate that cows avoid lying repeatedly on the same side for longer periods. The differences in lying side preference may be useful for assessing welfare and comfort. 

## 6. Factors Affecting Lateralisation Manifestation

The hemispheric specialisation pattern mentioned was considered a fundamental template that was conserved throughout vertebrate evolution, in which several factors could affect and modify this fundamental lateralisation pattern like experience and genetic factors that can lead to new species evolution. Genetics and experiences may have played a role, resulting in species-specific differences with unique lateralisation expressions compatible with the fundamental pattern. Therefore, depending on lateralisation ontogenesis, animals can perform lateralisation with varying degrees, or no lateralisation at all [17].

For example, the ability of sensory lateralisation to affect performance differs between species, as cattle were better than chickens in the odour discrimination task [145,146]. In a separate study, sheep and goats performed better than pigs in visual learning [147,148]. The visual acuity (i.e., the minimum resolution cycle per unit visual angle) was 1.3–2.5 cycles in cattle, 2.6–14.0 cycles in sheep, 2.9–3.1 cycles in goats, and 18.4–23.3 cycles in horses [149]. Further, motor lateralisation had been found to be species-specific [150]. These may be attributed to the anatomical differences between species, as in some species, the physical capabilities may limit the display of lateral preference. Furthermore, the deliberate selection of the flight response (left side handling/right hemisphere) traits in equid breeds may have affected the lateralisation manifestation, which reflects the strong relationship between lateralisation and emotionality [136,151].

Despite the strong evidence of the manifestation of motor or sensory lateralisation, it should be considered as a degree rather than as an absolute [64], since a higher degree of individual variation was observed in the lateralisation strength in most species studied [62,152]. There is also a relationship between the difference in lateralisation with individual variation shown in individual characteristics and behaviours [153]. Thus, individual differences could exert an impact on the measurement and degree of lateralisation. 

Age can also play a role in the degree to which lateralisation manifests. In this sense, evaluating lateralisation in adults in various species, including humans, faces several challenges due to various factors affecting asymmetry. However, studying neonates allows for many of these factors to be eliminated [154]. For example, the sensitivity to noise varies with age in heifers and bull calves, who have shown to react quicker to new sounds than cows and bulls [155]. Several studies have reported that age and individuality play an important role in posture lateralisation. For example, older cattle showed an increase in lying on the right side compared to younger cattle [143,156]. Arave and Walters [143] also reported increased lying on the right side with age advancing but not with increased parity. 

Furthermore, individual temperament can also affect sound sensitivity [155]; however, the performance of the individual during a detour task can be affected by its sex as suggested by the authors of [157], who stated that females showed higher motor lateralisation when compared to males.

When considering posture lateralisation, it is sometimes influenced by additional internal and external factors. With regard to internal factors, rumen fill, comfort, rumination, and pregnancy can affect behavioural lateralisation [158]. The study by Wagnon and Rollins [158] speculated that more time was spent by cows on the left side after feeding to support the rumen weight, which is situated on the left side. Furthermore, the lying side chosen by the individual can be influenced by comfort condition, as seen in ruminally cannulated dairy cows who spend less time on their left side (30%) when compared to intact cows (53%) due to the discomfort condition caused by the cannula [159]. Pregnancy can also affect cow lateralisation preference in that pregnant cows showed their preference for left side lying during advanced stages of pregnancy [153], especially for those carrying twins as opposed to singletons [144]. This could be due to the discomfort of the enlarged uterus that pushes the rumen more to the left. Furthermore, Mattachini et al. [160] deducted that primiparous cows lie more on the left side than the right side. In contrast, Tucker et al. [62] reported that pregnant cows at mid and late lactation, housed either in pasture or free stalls, do not have a lying side preference and that only the pregnant non-lactating cows showed a preference for the left side, which demonstrates the effect of the stage of pregnancy on lying side preference. 

External factors include differences between farming systems, in which pasture-based dairy cows showed equal distribution between sides in lying behaviour. However, the intensive farm-based cows displayed more lateralised lying preferences [23]. On the other hand, it was reported that lying laterality does not differ between varying levels of hock injury or lameness severity [142]; in another study, more left side lying was found in cows affected with mastitis [152]. Also, in one study, there was a left side lying preference [161]; in contrast, another stated a right-side preference [162] in cattle affected with bovine respiratory disease. The difference in lying side in cattle affected with bovine respiratory disease may be attributed to pain-associated discomfort. Furthermore, both studies had different procedures of inducing infection with *M. haemolytica*, as the authors of [161] inoculated the infection at the tracheal bifurcation (which should allow for an even distribution of the inoculum across both lungs), while the authors of [162] inoculated it in the right lung lobe in calves. Additionally, Černý et al. [163] revealed seasonal variations in the lying side of dairy cows, in which the number of cows lying on the left was 519 in the autumn and 444 in the spring, while the right side was 415 in the winter, and 320 in the autumn.

## 7. Relationship between Behavioural Lateralisation and Farm Management Practices

Husbandry, handling, and management can also affect the lateralisation of livestock. For example, cows that are raised under farming conditions are exposed to uncomfortable and distressful conditions starting from birth to sell [164,165]. Most of these procedures during this time are focused on efficiency, production, and maximising profit rather than on cow wellbeing [166]. Dairy herds, for example, are often milked twice or three times a day, a routine that has an important role in cows’ welfare, as their behaviour and physiology are adapted to it [167]. The demonstration of a preference for a certain side in the milking parlour is considered part of this milking routine for dairy cows. 

The ability of cows to determine the side of the milking parlour to enter has been considered an indicator of visual lateralisation, since it depends on the strength of the perceived threat, or due to the crowding effect before being milked. This can be used as a measure to differentiate between different individual characteristics of cows under routine situations, anxiety, or as a coping strategy with the milking place and process. It also reflects the emotional differences in the cow’s ability to cope with this environment, since they are unable to choose how to enter the parlour due to the crowded environment [168].

Previous studies have shown that some cows have a consistent side preference. In a study by Hopster et al. [169], they collected data from 89 cows during 28 consecutive months and deducted that 25.8% of the cows in >75% from the milking sessions had a side preference. In another study, Hopster and his co-workers [169] chose eight cows from those who showed a consistent side preference and another eight who were inconsistent with their side preference. They revealed that the proportion of cows that showed a side preference in the milking parlour persists over time, despite changes in group size, lactation stage, and season of the year. However, Tanner et al. [170], who worked on a herd of 1379 cows milked on 90 milking sessions, reported that there was no side preference found in 741 cows (53.7%), whereas 46.3% showed a side preference. 

It has been assumed that cows are more disturbed when milked on the unpreferred side of the milking parlour, which can lead to poor welfare. Zucs et al. [171] found a decrement tendency in the milk yield of cows milked in the unpreferred parlour side. In addition, Hopster et al. [169] revealed a negative effect on the latency to enter, milk yield, and heart rate when cows were being milked in the left side of the parlour (i.e., cows were considered to be experiencing discomfort). However, Paranhos Da Costa and Broom [172] concluded that there is no evidence of discomfort or stress in cows when milked in the unpreferred milking parlour side. 

Milking side preference and behavioural lateralisation could also reflect previous aversive interactions with humans or cows, which means that the response to challenges or threats in the milking parlour have an important impact on behaviour. Hansen and Damgaard [173] suggested that the coping strategies depend on the differences in predictability and controllability of the environment. Moreover, Prelle et al. [168] reported that cows that had a consistent milking side preference in the parlour were more fearful under novel situations and dominating over others in the access to resources. Previous studies have also reported that cows who demonstrated a left-side preference in motor functions, like lying [174,175] and passing a novel person [110], produced more milk. However, a high body condition score was found in cows using their right eye during fighting [10].

Visual lateralisation has also manifested in other farm management practices and human–animal interactions, leading to implications for animal welfare [117]. The levels of fear and/or aggression manifested by animals could be reduced in case of handling from the correct side. For example, Rizhova and Kokorina [176] concluded that the direction of feed delivery to cows on a feed belt affected their reproductive success and milk production. They reported that cows who received feed from their left side (i.e., left eye/right hemisphere) every day for several months had increased reproduction success and milk yield in comparison to cows that received feed from the right side (i.e., right eye/left hemisphere) every day. Furthermore, visual lateralisation effects on milk yield depend on the feeding conditions of the herd, with the left-side feed presentation increasing milk production under good feeding conditions and the right-side presentation increasing milk production under poor feeding conditions. This study suggested a relationship between the positive stimuli (feed) processing and the somatic processes controlling reproduction in the right hemisphere. 

Unlike cattle, horses are often handled and managed as individuals rather than a herd, resulting in more human–animal interactions than seen in many other livestock species. As a result, the link between direct human interaction and lateralisation choice of horses can provide additional indicators of the welfare of horses within the interactive experience. Since horses are often trained and handled from the left side, most scientific experiments focused on behavioural responses often set up the experiments or specifically train horses to compensate for any side bias. Therefore, many of the research studies on equine side preference eliminate lateralisation from the design. A few, however, like the cognitive tests listed previously, looked specifically at lateralisation as a means of assessing the emotional welfare of animals in the domestic environment. Additionally, few studies have addressed lateralisation under saddles, where sidedness is often deterred. One study, however, looked at the lateralised responses of horses to patting or scratching by their rider (two forms of physical contact often used by riders to “reward” the horse for good behaviour). In this study, horses displayed more left ear lateralisation during neck patting, which was associated with higher stress levels, and a right ear lateralisation (right ear forward) during wither scratching, which was associated with lower stress levels and lower heart rate variability [177].

Therefore, informed, and deliberate lateralisation can provide a practical application for improving farm animals’ welfare, in addition to financial profit to the farmers.

## 8. Relationship between Behavioural Lateralisation and the Infrared Thermography (IRT) Technique

Animals often adapt their behaviours under challenging and uncertain conditions, potentially even displaying behaviours that do not accurately represent their true emotional state. Under these circumstances, increased or intensified handling can elevate stress responses and intensify their reactions. Therefore, it would be more useful to have some other effective assessments that linked behavioural responses with physiological changes. In this context, there is evidence of a relationship between anxiety and variation in body temperature of both cows and horses. For example, Lees et al. [178] reported that the temperament of beef cattle influenced the regulation of its body temperature, while in another study [41], researchers observed that the pharmacological induction of anxiety in beef cattle leads to higher rectal temperatures than non-anxious cattle. 

**Figure 1:**
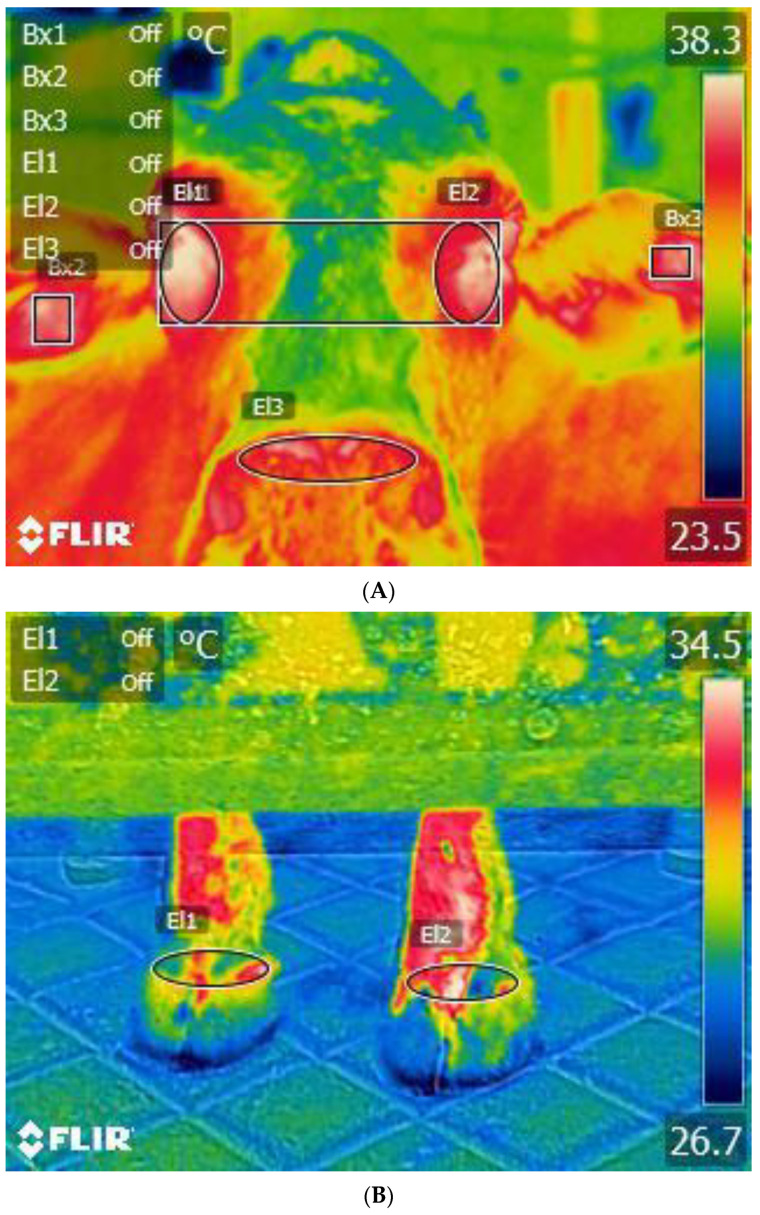
Front-on-view thermograms of a cow’s head (**A**) and lower forelimbs (**B**). Adapted from [60]. In A, El1 and El2 indicated the external surfaces of the right and left eye, respectively. Each eye area included the region extending from the outer to inner canthus of the particular eye, including the orbital and periorbital area. Bx2 and Bx3 indicated the external surfaces of the right and left ear pinna, respectively, along with the artery and vein. El3 indicated the external surfaces of the muzzle region above the nostrils. Similarly, in B, El1 indicated the external surfaces of the right coronary band of the forelimbs, and El2 is of the left. The coronary band of the forelimbs included the thickened band of extremely vascularised tissue that lies above the upper edge of the particular coronary band of the forelimbs. Triangle marker in each body part indicated the pixel with maximum infrared temperature. The coloured index scale shown on the right side of the thermograms represents the highest, lowest, and intermediate infrared temperatures with the hues of white, blue, and others; red, yellow, and green, respectively.

However, the estimation of rectal temperature requires handling and restraining, a method which may incur stress and influence results. Therefore, in order to reduce potential stress, data collection for temperature can be completed by reducing handling or insertion of equipment into the body or using devices that are implanted in the animal using minor surgery. Although surgical implants can be helpful, these devices may cause pain and distress, and they are impractical for many commercial herds due to cost, the need for skills in implantation, and the risk of infection [179,180]. Thus, it is still challenging for animal stakeholders to effectively identify these subtle temperature changes linked to behavioural lateralisation and distress. In this aspect, researchers have shown that infrared thermography (IRT) could be a low-stress option to identify these subtle changes in the body temperature remotely and non-invasively. Infrared thermography measures the radiated heat from the external body surfaces such as the eyes and coronary band of limbs (Figure 1) in proportion to the changes in the core body and peripheral temperature, respectively [30,60,61,181,182,183]. 

Several studies have investigated the relationship between IRT measures and respective behavioural lateralisation. Uddin et al. [60] investigated the relationship between the IRT of cows’ external body surfaces and a range of lateralised behaviours, including the side (left or right) of passing a person in the lane, sniffing the ground, stopping while walking, rumination, tail and ear movement, and ear and head position. They reported that the maximum IRT from the captured thermogram was more associated to the behavioural lateralised responses of cows than the average IRT. They also found that cows that favoured the right over the left side when passing the person in the lane (right lateralised/right hemisphere) had a higher limb and eye IRT. In contrast, Uddin et al. [60,183] found no difference in the IRT of the left and right eyes. However, the maximum IRT of the right eye showed a stronger association with the behavioural measures than that of the left eye [60]. They also reported a positive association between the IRT of the left eye and the IRT of the right eye with the right side passing (right lateralised: more anxious cow) and stopping while walking in the lane (hesitating behaviour) when passing an unfamiliar person after the evening milking. Despite these findings, the IRT of the right eye was found to be positively associated with the daily milk yield as well as the head-down position while walking in the lane, while the IRT of the left eye was negatively associated with milk fat content. The same pattern of relationship (stronger on right eye than left eye) was also observed in a comparison between the left and right eye’s average IRT when combined with laterality. The right-lateralised cows showed a higher IRT in the right eye than the left-lateralised cows, while the left eye’s IRT did not differ between the right- and left-lateralised cows [60].

Uddin et al. [61] again reported a similar association between the eye and limb IRT with the lateralised behaviours. They also found that the IRT measures showed a higher association with the lateralised behaviours than rectal temperature. In addition, the IRT measures, particularly the eye IRT, were repeatable, while the rectal temperature was not. Estimation of cows’ external body surface’s IRT needs more consideration of the temperature humidity index, days in milk, and somatic cell counts in milk as confounding factors. For more details, see Refs. [60,61,183,184]. 

IRT has also been used in horses. Non-invasive measures of infrared thermography of the eyes in horses has been successfully used to determine stress levels in horses during performance [55], human handling [50,56], and interactions with the riding equipment [57]. More studies are needed, however, on the link between the IRT in horses and their lateralisation preferences. 

## 9. Conclusions

Research in lateralised behavioural responses to environmental stressors is becoming more common as a means of improving the evaluation of livestock welfare. Recently, research has created a scope for the non-invasive and remote evaluation of stress responses in animals using infrared thermography. Although more research needs be conducted using different species, the combined implementation of both behavioural lateralisation and infrared thermography as indicators of animal preference and emotional valance are effective and accurate ways to easily assess and improve animal welfare in farm environments. 

## Data Availability

Data sharing is not applicable to this article.
